# Preliminary Analysis of Burn Degree Using Non-invasive Microwave Spiral Resonator Sensor for Clinical Applications

**DOI:** 10.3389/fmedt.2022.859498

**Published:** 2022-04-05

**Authors:** Pramod K. B. Rangaiah, Bappaditya Mandal, Erik Avetisyan, Arvind Selvan Chezhian, Bobins Augustine, Mauricio David Perez, Robin Augustine

**Affiliations:** ^1^Ångström Laboratory, Department of Engineering Sciences, Microwaves in Medical Engineering Group, Solid State Electronics, Uppsala University, Uppsala, Sweden; ^2^Ångström Laboratory, Division of Computer Systems, Department of Information Technology, Uppsala Networked Objects (UNO), Uppsala University, Uppsala, Sweden

**Keywords:** loop probe, flexible sensor, microwave sensor, spiral resonator (SR), circuit analysis

## Abstract

The European “Senseburn” project aims to develop a smart, portable, non-invasive microwave early effective diagnostic tool to assess the depth(d) and degree of burn. The objective of the work is to design and develop a convenient non-invasive microwave sensor for the analysis of the burn degree on burnt human skin. The flexible and biocompatible microwave sensor is developed using a magnetically coupled loop probe with a spiral resonator (SR). The sensor is realized with precise knowledge of the lumped element characteristics (resistor (R), an inductor (L), and a capacitor (C) RLC parameters). The estimated electrical equivalent circuit technique relies on a rigorous method enabling a comprehensive characterization of the sensor (loop probe and SR). The microwave resonator sensor with high quality factor (Q) is simulated using a CST studio suite, AWR microwave office, and fabricated on the RO 3003 substrate with a standard thickness of 0.13 *mm*. The sensor is prepared based on the change in dielectric property variation in the burnt skin. The sensor can detect a range of permittivity variations (ε_*r*_ 3–38). The sensor is showing a good response in changing resonance frequency between 1.5 and 1.71 GHz for (ε_*r*_ 3 to 38). The sensor is encapsulated with PDMS for the biocompatible property. The dimension of the sensor element is length (L) = 39 mm, width (W) = 34 mm, and thickness (T) = 1.4 mm. The software algorithm is prepared to automate the process of burn analysis. The proposed electromagnetic (EM) resonator based sensor provides a non-invasive technique to assess burn degree by monitoring the changes in resonance frequency. Most of the results are based on numerical simulation. We propose the unique circuit set up and the sensor device based on the information generated from the simulation in this article. The clinical validation of the sensor will be in our future work, where we will understand closely the practical functioning of the sensor based on burn degrees. The senseburn system is designed to support doctors to gather vital info of the injuries wirelessly and hence provide efficient treatment for burn victims, thus saving lives.

## 1. Introduction

Around 180,000 deaths annually occur due to burn injuries, making it one of the global health problems (1). Almost two-thirds of cases occur in low-income areas in Africa and South-East Asian regions (2). The hospital cost of burn management exceeded *e*10.5 million for Norway in 2007 (1), Kenny Asselin (3). Every year over 450,000 serious burn injuries occur in the United States that require medical treatment. According to American burn association report, in 2014 alone, there were 3,275 recorded deaths from fire and smoke inhalation injuries. According to a report from the Agency for Healthcare Research and Quality in 2016, the total cost for the treatment of burns in 2010 was $1.5 billion, with another $5 billion in costs associated with lost work (3). The research on burn injuries and analysis is becoming essential since unintentional death and damage cases increase. Research activities related to the burnt depth and degree study give scope to unique projects worldwide (4). There are many causes for burn cases related to fire flame, hot object contact, chemicals, electric shocks, etc. Early and proper analysis of burnt depth helps the doctor to choose the right treatment. Burns are generally categorized based on the depth of injured skin and can be typed as (i) first degree (superficial), (ii) second degree (partial-thickness), and (iii) third degree (full thickness) (5). In this work, we have generated a significant contribution toward the MedTech research project known as “Senseburn” http://www.senseburn.com/, and it is funded by EU Eurostars program to design an innovative burn analytical instrument for scientific use in the medical field (6) for analysis of burn depth(d) using a non-invasive microwave sensor and to automate the complete process and display 3D human body models (7).

The dielectric spectroscopic classification at microwave frequencies and color profiling of *ex-vivo* human samples was done in our previous research (4). Furthermore, the microwave sensor is developed based on the microwave reflectometry technique to analyze the burnt degree analysis (8). As part of the senseburn project, the dielectric profiling of the healthy and burnt skin variation with saline injection was conducted (9). Our previous published work illustrates its proof of concept. It is feasible to differentiate various burn depths by their corresponding dielectric profiles. However, some additional prerequisites have to be collected to ensure the findings are statistically consistent. The dielectric profile of the samples is equivalent to the superficial dermal burns (typical dry skin values). More profound injuries show lower dielectric profiles, reaching values similar to those corresponding to bone and fat. The findings from our previous experiments reveal that a novel prototype for the microwave sensor using the loop probe and spiral resonator (SR) has been developed in this work.

There are several works done on microwave sensor such as Dash et al. (10) designed a microwave sensor using a dual-band circularly polarized triangular dielectric resonator antenna. The rectangular shape resonators are employed with narrow copper strips. Benkhaoua et al. (11) designed Edge-Coupled SR for organic tissue biosensing application, which is useful, especially in the treatment related to microfluidic liquids and organic tissues of the human body. Also, SR exhibit sharp resonance and detect water thickness effective permittivity in the material by changing resonance tangible frequency (12). Chen (13) experimented with magnetically coupled microwave sensors for wearable and implantable applications using passive loop antennas. Shafi and Abou-Khousa (14) developed the microwave sensor using a small loop antenna loaded with SR. The resolution of sensing is up to 5 mm, and it is used to detect defects in the composite structure. Eldamak and Fear (15) designed the disposable sensor for the hydration monitoring based on the sweat data. Griffith et al. (16) used EM resonator based sensor for monitoring intracranial pressure in the brain. Cluff et al. (17) designed an open circuit EM resonant sensor that can be placed on the skin which will illustrate the relationship between fluid volume and resonant frequency.

This article is a preliminary study in view of fabricating the microwave sensor for measuring burn degree (clinical application). The focus in the article is on drawing information by correlating simulations to our previous studies (4). This work provides a convenient approach of using microwave resonator based sensor to assess the burnt human skin analysis. The proposed sensor can be used directly on the burnt patient. Also, the system is made portable and user friendly for operation. This paper is organized as follows. Section 2 provides detailed descriptions of the material and methods used in this work. Section 2.1 describes the relationship between permittivity and burn human tissue variation. Section 2.2 explains the development of the senseburn sensor. The detailed estimation of the RLC parameters of the sensor is explained in Section 2.3. Section 3 explains the important results and a detailed discussion of the burn degree analysis. The sensor is made bio-compatible and flexible for the clinical trial. The application details are explained in Section 3.5. Conclusion and future work is described in Section 4.

## 2. Materials and Methods

### 2.1. Effective Relative Permittivity vs. Burn Degree

**Figure 1:**
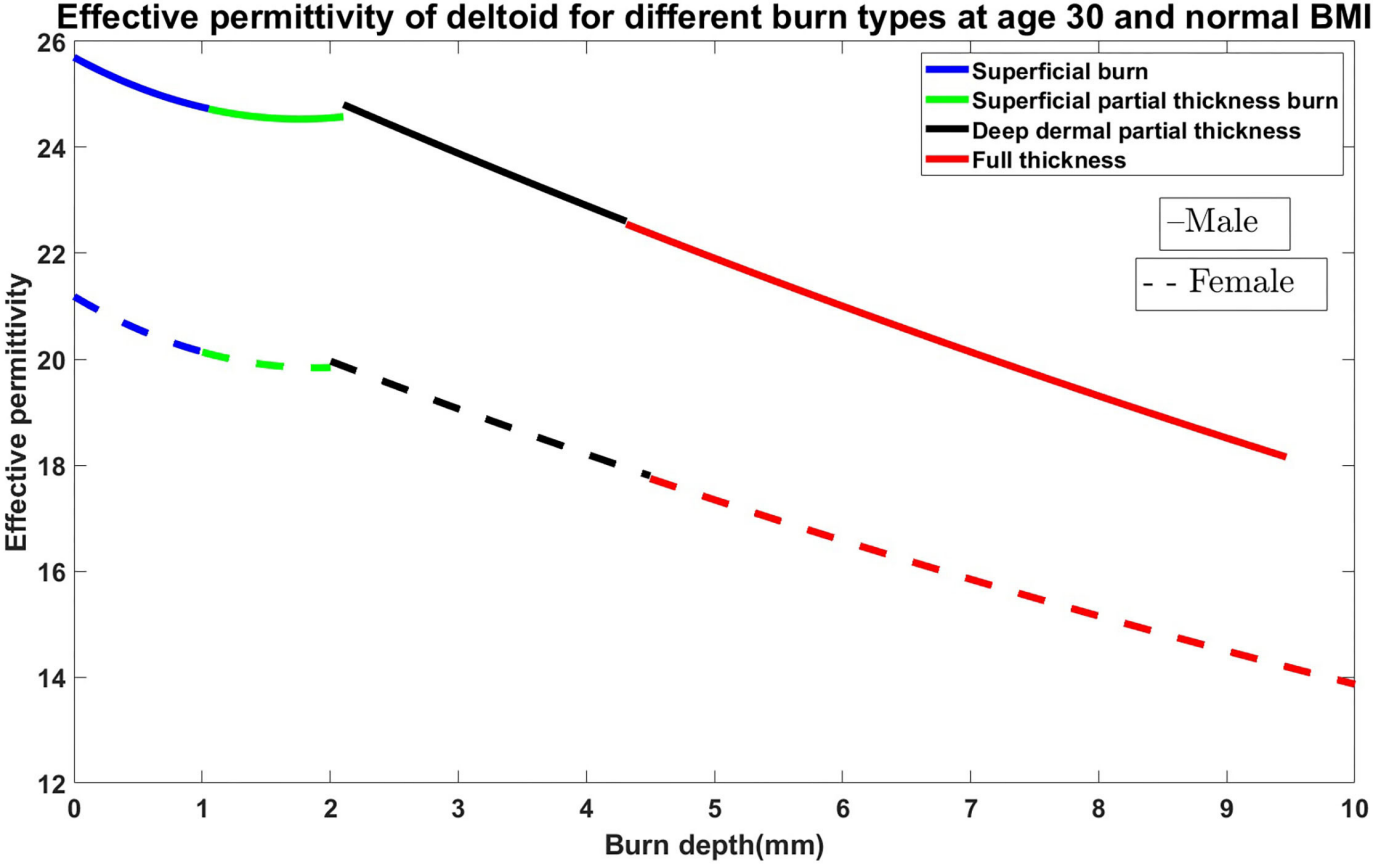
Effective permittivity of the burned skin vs. burn degree for the case of 30 years old male and female on the deltoid region with normal BMI.

In this section, the relationship between permittivity and burn degree based on the fundamental parameters such as age, body mass index (BMI), gender, and body parts are presented. The simulation is carried out using MATLAB. For the analysis of burn degree, finding the thickness of the human tissue layers in the particular body parts is very important. We considered skin (epidermis and dermis), fat, and muscle thickness for the evaluation of the burn degree. Factors affecting human tissue layers are age, gender, BMI, and anatomical location of the body (18). The dielectric properties of the *in-vivo* epidermis and dermis with temperature consideration are used in our simulation based on the research data by Sasaki et al. (19). The algorithm was chosen to have an average thickness of the skin and subcutaneous adipose fat tissues based on gender and BMI data extracted from the experiment conducted by Akkus (20) is considered in the algorithm. Estimation and relationship between the skin, fat, and muscle layers based on the fundamental parameters are given in Shankar et al. (21), Bueno et al. (22), and Mechelli (23). Similarly, the relationship between muscle and fat has been studied by Welch et al. (24). The impact of age and gender on the muscle and fat layers, and various body parts are investigated (25, 26). Papp et al. (27) developed the reproducible model for the burn samples in the pigs. Later, Papp et al. (28) performed sensitive and non-invasive methods to evaluate dielectric parameter measurements for superficial, partial-thickness, and full-thickness burn. Our previous works (29) verified permittivity variations in various *ex-vivo* burnt human skin samples. Rangaiah et al. (4) made efforts to cluster the burn variations based on the permittivity values and experimented with edema tissues considering differential partial and full-thickness burn cases. The relative changes in the dielectric properties of the *in-vivo* burned are of the skin in relationship with the degree of burn is done by Oppelt et al. (30). Several articles and website information are considered to prepare the efficient algorithm to decide the average thickness of the human tissue layers. Figure 1 shows the effective permittivity of the burned skin vs. burn degree for the case of 30 years old male and female on the deltoid region with normal BMI. Similarly, the algorithm is made for age between 1 and 100 years; BMI with low, normal, obese, and overweight; Gender: male and female; different anatomical regions: deltoid, thigh, abdomen, and suprascapular (31).

Permittivity describes the polarization of isolated material when the electric field (E-field) is applied. There are different types of permittivity, the most used is the relative permittivity or dielectric constant, i.e., the permittivity relative to permittivity in a vacuum, which is equal to 1. The relation between the permittivity and permittivity in a vacuum is described in equation (1), where χ_*e*_ is electric susceptibility.


(1) $$\begin{array}{l} \epsilon_{r}=1+\chi_{e}. \end{array}$$

The permittivity of given material changes depending on measurement frequency. Italiano National Research Control Department of applied physics (IFAC) in Florence created an application based on the parametric model for the calculation of the dielectric properties of body tissues (32). Data for skin, subcutaneous fat (SF), and muscle were taken from IFAC application and the skin was assumed to be dry. The devices for burn diagnostics and sensors were designed for the frequency 2.45 GHz; hence, the permittivity of the values taken from IFAC was also for that frequency.

Data given in IFAC is for each separate tissue, and the data for multi-layered tissue is not available on that web page. When the phantom contains several layers, it is common to use the concept of effective permittivity, which is the combined permittivity for all tissues. There are several models to find the effective permittivity. During this work, Maxwell Garnett approximation with shape distribution (SDMGA) was used to combine the relative permittivities of skin, SF, and muscle tissues. Maxwell Garnett approximation is a mathematical model for effective medium approximations (EMA) and this model is effective for a homogeneous medium. Equation (2) illustrates the Maxwell Garnett approximation


(2) $$\begin{array}{l} \epsilon_{e}=\epsilon_{2}\frac{1+f\left(\sqrt{\epsilon_{1}/\epsilon_{2}}-1\right)}{1+f\left(\sqrt{\epsilon_{2}/\epsilon_{1}}-1\right)} \end{array}$$

where ϵ:s are relative permittivities, for two different layers, ϵ_*e*_ is the effective permittivity of two combined layers, and *f* = *h*_2_/*h*_1_ where *h*_2_ and *h*_1_ are layer thicknesses (33).

Maxwell Garnett approximation with shape distribution is adapted for two layers with different thicknesses and permittivities, but in this work, three layers need to be examined which means that SDMGA will be used two times in the same calculation. To mimic the permittivity and thickness change in tissues, the water concentration is decreased in the program by defining the amount of water that the tissues contain and decreasing it by one percent at a time. At the same time, the thickness and permittivity of the tissues were also decreasing linearly with water concentration and the results were plotted in thickness range, depending on how much water was lost because of heat.

The superficial partial-thickness burn is a second-degree burn that damages the epidermis and the upper parts of the dermis. A higher amount of water is affected due to deeper burn and the electrical properties are affected even more (34). The skin contains about 80% of water, and it was assumed that the superficial burn decreases the water concentration by 50%, and the thickness of the skin layer was decreased by 40%. Therefore, the percentage of water concentration was varied between 0 and 50%, and the skin thickness was decreased from 0 to 50%. Blisters and swellings are not related to superficial burns. Sunburn can also be classified as superficial burn (34). It was assumed that SF and muscles were not affected and either the thickness or water concentration in these tissues were changed. The relative effective permittivity for the total tissue was calculated by using the thickness and permittivity of the healthy skin, SF, and muscle. For the superficial partial-thickness burn, the remaining 40% water was decreased from 50 to 100% and the skin thickness was reduced from 40 to 80%. It was assumed that the thickness and permittivity of the SF and muscle were not affected, and the relative effective permittivity for the total phantom was calculated by the relative effective permittivity of the SF and muscle and later using that value in SDMGA again in combination with the skin thickness and permittivity. For the deep partial thickness burn, SF contains less water than muscles and skin. The SF contains approximately 10% water, and it was assumed that for a deep partial-thickness burn, the water concentration will decrease from 0 to 50% and the thickness of the SF will decrease from 0 to 0.05%. To find the relative effective permittivity, the SDMGA was used only once because the only healthy tissues left were part of SF and the total muscle tissue. The full-thickness burn can even affect the muscles and bones, but during this simulation, it was assumed that the muscle tissue is not affected. To simulate the full thickness burn, water concentration was reduced from 50 to 100% in SF tissue and the thickness of SF tissue was reduced from 0.05 to 0.1%. SDMGA was used only to calculate the relative effective permittivity of the lower part of the SF and healthy muscle tissue. It was also assumed that the bone permittivity does not affect the relative effective permittivity.

Numerous analyses were made to find the relationship between the burn variations concerning permittivity. Based on this analysis, we started developing the sensor for the evaluation of burn degree. The sensor should be able to detect the permittivity variations on the human skin and it should effectively detect the small variations in terms of the shift in resonance frequency.

### 2.2. Sensor Development

This section delivers the technical details of the sensor prototype optimized for burn degree. Many different noninvasive imaging techniques have been investigated for assessment of burn degree and have been reviewed in detail Kunjachan et al. (35), Giakos et al. (36), Murray et al. (37), Balaban and Hampshire (38), and Schoenhagen et al. (39). The most successful of these technologies involves an imaging modality that measures blood flow. However, we still need improvement for its use in burn wound detection because of long acquisition times and high costs. Open circuit resonant sensors can be designed as wearable, soft bioelectronics, similar to a skin patch that can be applied as an adhesive bandage, allowing it to be used as a point-of-care diagnostic technology to detect burn degree. It do not require electrical components and are passively energized using an external radiofrequency (RF) sweep. Based upon the premise that impedance fluctuations arise as a result of fluid volume changes, open circuit electromagnetic skin patch sensors may be able to detect changes in water content in the skin, meaning changes concerning dielectric constant. Dielectric changes inside a human skin can be identified as a shift in the resonant frequency of the open circuit resonant sensor skin patch. The objectives of this study were to develop a point-of-care, noninvasive electromagnetic resonant skin patch sensor to detect the burn degree.

**Figure 2:**
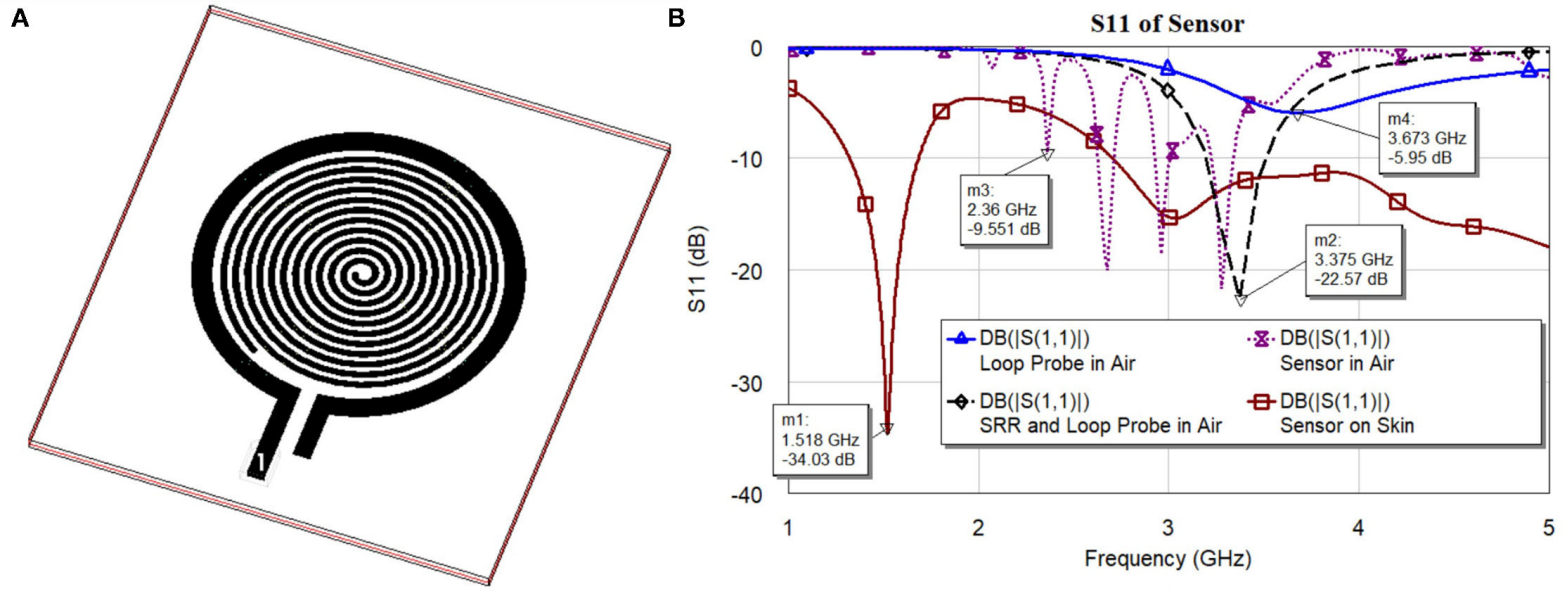
**(A)** 3D Orthogonal view of the sensor and **(B)** *S*_11_ characteristics of the sensor and its elements.

To develop the third prototype sensor for burn degree analysis, a magnetically coupled loop antenna with an SR as shown in Figure 2A was chosen. For sensing technology, we required a high quality factor (Q) with a narrow band. Here, we developed a microwave resonator sensor with high Q on the Rogers RO 3003 substrate with a standard thickness of 0.13 *mm*. The electromagnetic resonant sensor patch was designed and built from a single baseline component: a trace of copper, which was configured into a circular planar spiral. The SR and loop probe [circular spiral patch, (radius 14*mm*)] had 12 turns as shown in Figure 2A. The detailed geometry with RLC parameter analysis is discussed in Section 2.3.

Figure 2B shows *S*_11_ characteristics (resonance frequencies) of the sensor and its elements. The sensor is made of two major elements, namely loop probe and SRR. To explain the sensor operation, every element is simulated separately and is discussed. In the figure legend, the *S*_11_ curves are marked with a loop probe in Air, SRR, and loop probe in air, sensor (loop probe and SRR encapsulated with PDMS), and sensor on human skin model. The dielectric definitions at 2.45 GHz of the three-layer human tissue model includes skin (2 mm) ε_*r*_ 38 and dielectric loss tangent (tanδ) 0.2, fat (1 cm) ε_*r*_ 5.2 and tanδ 0.14, and muscle (2 cm) ε_*r*_ 52 and tanδ 0.24. The detailed evaluation of the sensor with RLC parameter analysis is explained in Section 2.3.

#### 2.2.1. Sensor Fabrication

**Figure 3:**
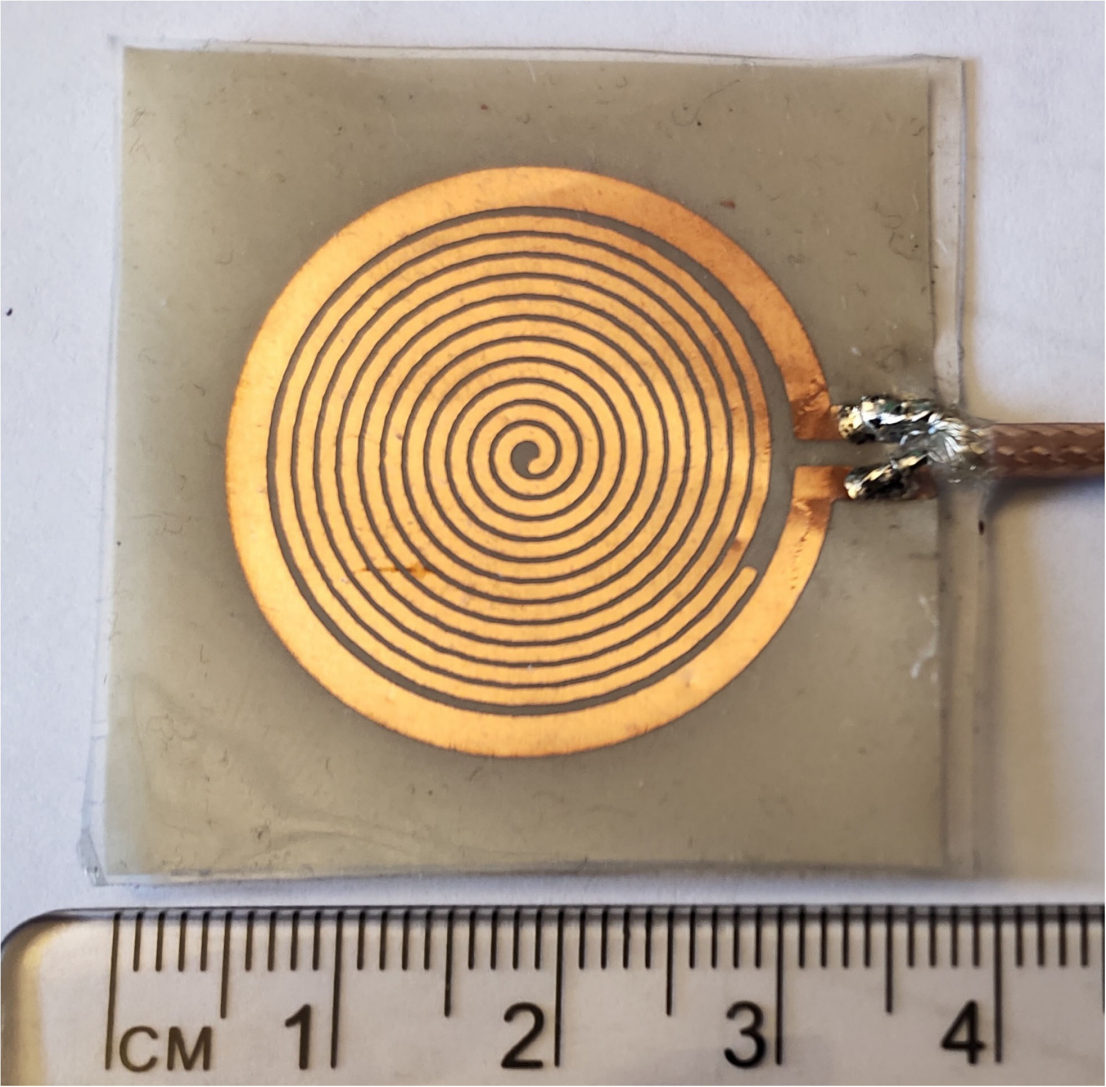
Sensor fabricated picture.

Figure 3 shows the picture of the fabricated sensor unit. The proposed sensor is fabricated on the standard RO 3003 substrate with a standard thickness of 0.13 mm. The substrate was having copper cladding on both sides, one side of the substrate is exposed to the UV through a photo-resist mask with the sensor pattern. Another side copper deposition is removed, which makes the sensor flexible. The fabricated sensor board is then encapsulated with PDMS (wacker silicones elastosil, 10:1 monomer, and curing agent), which gives mechanical stability, water resistance, inertness and bio-compatibility (40).

#### 2.2.2. Requirement for Flexible Sensors in Burn Analysis

The degree of burns can range from first to fourth. The first degree burns limits to the epidermis, second degree till dermis, third till subcutaneous tissue layer, and fourth the most severe, till muscle and bone. Gathering essential information from these types of burns using traditional sensors with rigid structures restricts the surface it can cover. It also creates non-uniformity in measurement as the device does not follow the natural contours of the body. It can also cause severe distress for the patient who is already undergoing serious trauma. The flexible sensors will allow conformity on the injury as they can be incorporated as skin patches and can also provide user comfort. It will provide homogeneous contact with the body part as it is compliant with the curves. This will help in keeping reference parameters consistent, providing also better coverage of the burn injury, and thus will result in accurate and reliable measurements for data analysis.

The sensors are flexible enough to be conformal and does not undergo large deformations to cause significant variation in measurements. The main interesting parts of burn analysis is body parts depending on the surface area. Majorly, size of the burn is estimated using rule of nines. It divides the body surface area in to percentages. It is distributed as head and neck (9%), chest and upper back (9% each), arm (9% each), abdomen and lower back (9% each), and genital area (1%). The flexible sensors are focused on to be placed on these major areas and large flexibility is not required in these parts in comparison to fingers or toes. But these sensors should be flexible enough to ensure conformity to the body surface, especially in the case if it could be applied underneath the bandage on the open wound. As this is a preliminary study, the understanding of conformally flexible sensors on crucial parts as mentioned earlier was focussed as scope of this work and characterization of any significant variation caused by sensors under extreme deformation would be explored in our future work.

The Rogers Duroid RO 3003 substrate offered a good solution for the proposed microwave sensor in terms of flexibility and lighter weight structure that is suitable to place on the human body. Also, Rogers RO3003™ has the property to produce an omnidirectional pattern since the human body can block backward radiation which is popularly known as the body shadowing effect (41).

#### 2.2.3. Reliability and Bio-Compatibility

In practical application, the sensor will be placed on the skin without damaging the tissues. No cable will be in contact with the patient or will be covered with a medical-grade sterilized plastic bag provided by the hospital. The sensor is encapsulated with a PDMS layer of approximately 1 mm thickness to avoid any inflammatory response. Sensor encapsulated with PDMS provides the least impact and interference on the burnt tissues (42). The fact that PDMS is bio-compatible and bio-stable makes it the most studied implantable polymer (43). Currently, PDMS has been used as a coating on a sensor that can be placed on both humans and animals. The period that the sensors are in contact with the human body are usually very short time (maximum of 5 min). PDMS is optically clear and in general, inert, non-toxic, and non-flammable (44). We have used similar sensors on the *ex-vivo* tissue samples without changing any properties or damaging the tissues. The medical devices encapsulated solely by PDMS lack the longevity for use in chronic implant applications due to defect-related moisture penetration through the packaging layer caused by conventional deposition processes such as spin coating.

### 2.3. Estimation of RLC Parameters of the Proposed Sensor Using Theoretical and Simulation Models

In this section, the detailed electrical characteristics of the proposed sensor have been explained. The proposed sensor is evaluated into two major microwave structures (loop probe and SR) on the same plane. First, the moment method charge distribution and distributed capacitance of SR is found using the simplified model. Later, loop probe and SR accurate RLC lumped elements estimations will be done.

#### 2.3.1. Evaluation of Distributed Capacitance of SR

**Figure 4:**
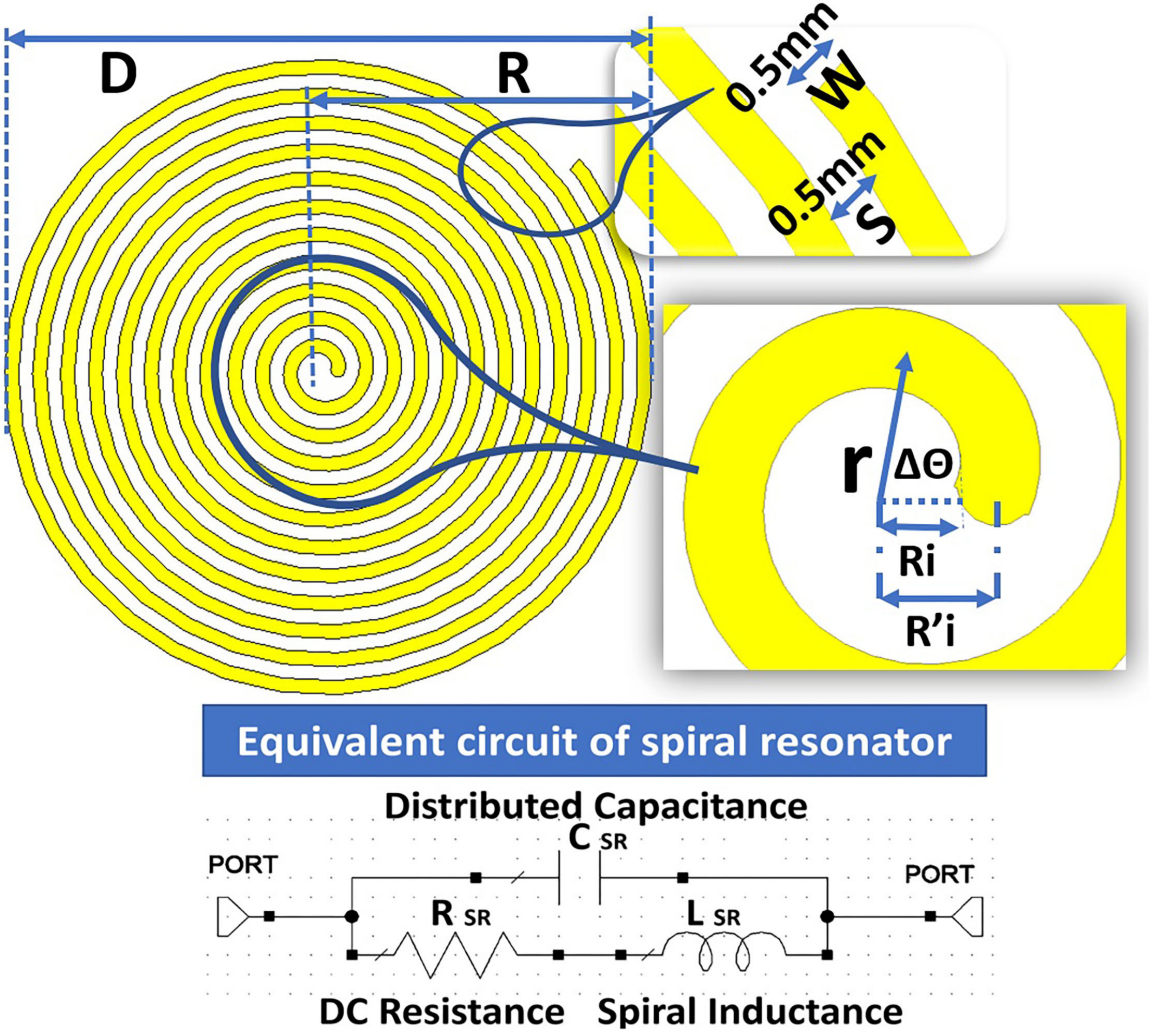
Structure of the spiral resonator (SR) with an equivalent circuit to calculate the distributed capacitance.

A planar SR system includes spiral rings on a substrate. For easy theoretical analysis, the spiral rings are considered to be in a uniform dielectric medium with permittivity having an equivalent circuit shown in Figure 4. The capacitance C_0_ per unit length of the SR can be found from the method described in Palermo (45) and the overall distributed capacitance C of the SR can be found (46).

An empirical formula with limited accuracy (47) to calculate distributed capacitance (C) in the planar spiral rings are expressed as in Equation (3)


(3) $$\begin{array}{l} C=0.035D_{0}+0.06\left[pF\right] \end{array}$$

Where *D*_0_ is the diameter of the outer ring expressed in millimeters. This expression is basic if we neglect many appropriate parameters.

The planar SR includes the spiral ring structures on the RO 3003 substrate with a thickness of 0.13 *mm*. For calculation, it is considered a uniform dielectric medium with permittivity ε = 3 and assumed capacitance C_0_ per unit length of the SR. The overall distributed capacitance C of the SR is calculated based on the width W = 0.5 *mm* of the spiral conductors and spacing S = 0.5 *mm* between the turns. The equation for the spiral is given as $r=R_{i}^{\prime }+\frac{W\theta }{\pi }$. $R_{i}^{\prime }$ is the primary mean radius equal to $R_{i}+\frac{W}{2}$, where *R*_*i*_ is the inner primary radius; r indicates the mean radius; and θ is the rotational angle in radians. The details of the parameters are given in Figure 4.

The voltage differences between ends of the SR with N (12) turns are represented by U. Now, voltage per turn is given by U/N and distributed capacitance of SR is expressed as in Equation (4).


(4) $$\begin{array}{l} C=\frac{q}{U}=\frac{1}{U}\int_{0}^{2\pi N}\frac{U}{N}C_{0}\left(R_{i}^{\prime }+W\frac{\theta }{\pi }\right)d\theta \end{array}$$

Therefore, the distributed capacitance C in relationship with outer radius R_0_, inner radius by assuming C_0_ is independent of r is given in Equation (5)


(5) $$\begin{array}{l} C=\pi C_{0}\left(R_{i}+R_{0}\right) \end{array}$$

**Figure 5:**
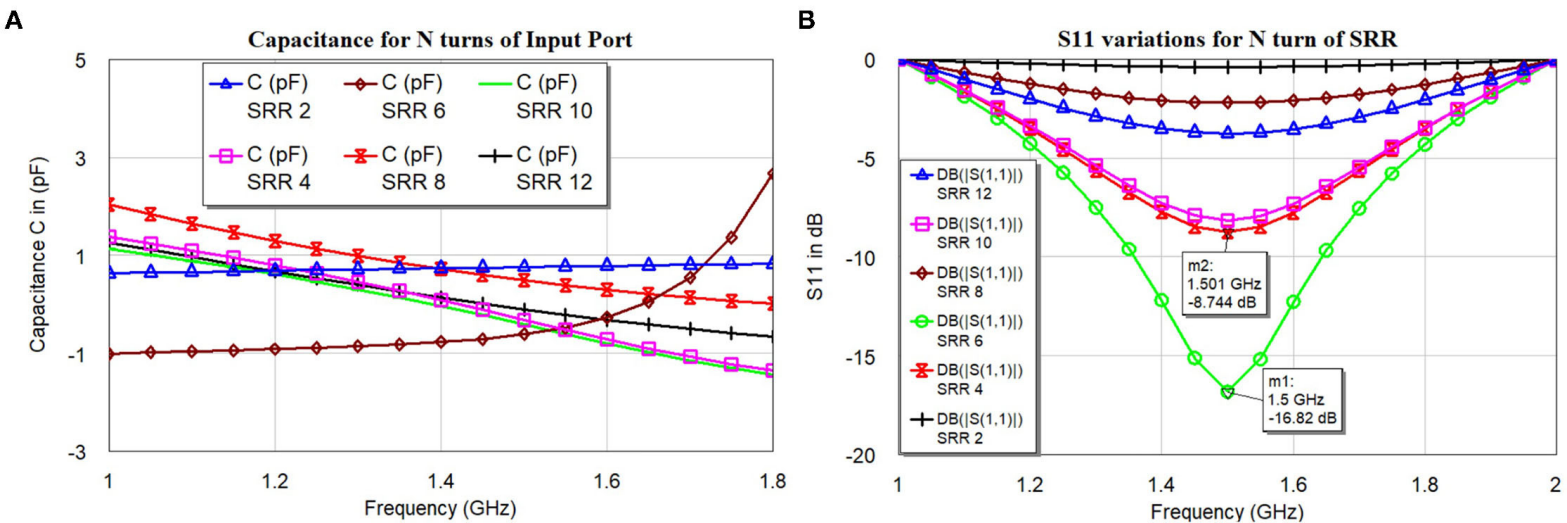
Number of turns in SR effects **(A)** Capacitance and **(B)** S_11_ variations.

The proposed SR is having an outer diameter (D) = 22.76 *mm*, outer radius (R) = 11.38 *mm*, inner diameter (D_*i*_) = 1.28 *mm*, and inner radius (R_*i*_) = 0.64 *mm*. The ratio between inner and outer diameter or radius $\frac{D_{i}}{D}=\frac{R_{i}}{R}=0.05$ and the ratio between width (W) and spacing (S) *W*/*S* = 1; the number of turns simulated are N = 2, 4, 6, 8, 10, and 12. The thickness of the dielectric medium with ϵ = 3, the substrate is t_*s*_ = 0.13 *mm*. The width W of SR is found to be $W=2.4*\frac{\left(D-D_{i}\right)}{4N+2}$. Figure 5B shows the results of simulated S_11_ for an even number of turns between 2 and 12. Figure 5A shows the corresponding capacitance values under the condition of constant of the inner and outer radius of the SR.

The distributed capacitance of the various turns is weakly dependent on the number of turns (N), this might have happened because of non-uniformity current distribution throughout the spiral rings. The current distribution will be more at the central part, the inter-turn capacitance will be treated as parallel capacitance as shown in Figures 4, 5A. The negative values of the capacitance can be treated as series inductance values with positive values, but it is not linear in the function. The simulated S_11_ (Figure 5B) indicates that resonance frequency is independent of the N turns but varies with the magnitude.

#### 2.3.2. Reliable RLC Parameters Extractions

**Figure 6:**
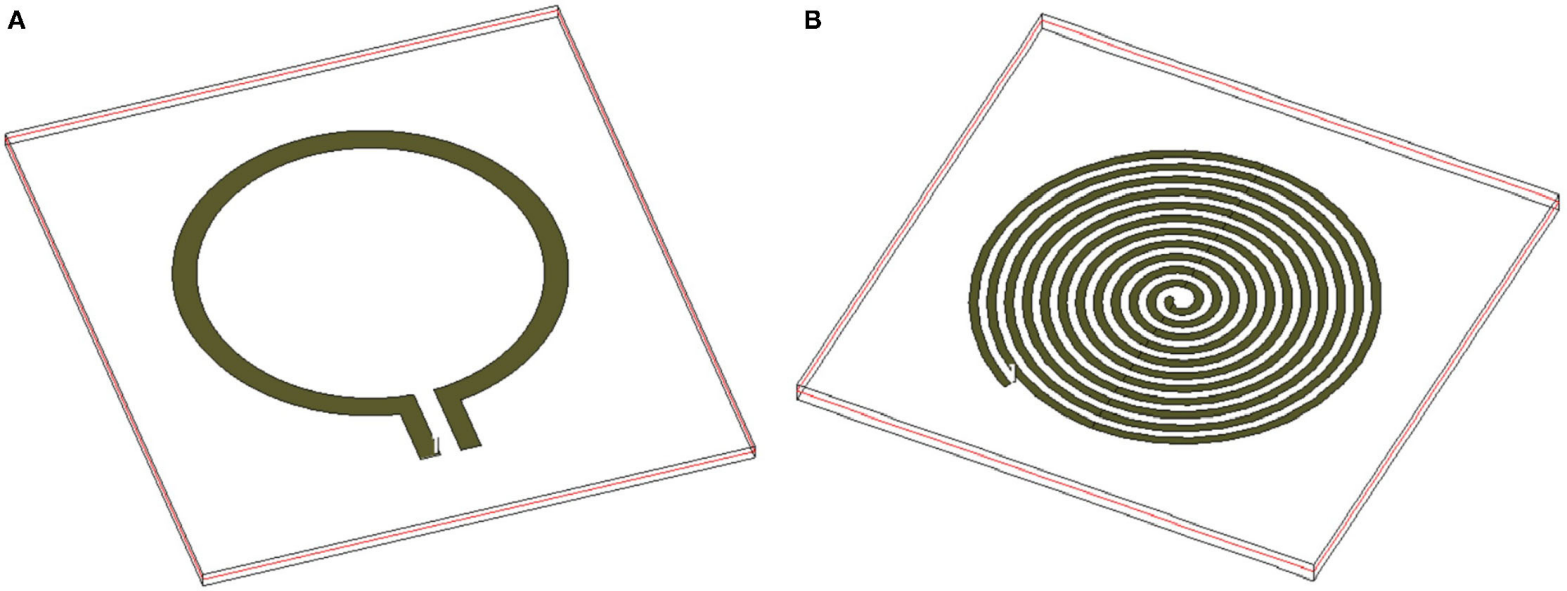
Major two microwave structures involved in the sensor **(A)** loop probe and **(B)** SR.

The sensor consists of two major microwave structures namely (a) loop probe and (b) SR shown in the Figure 6. The loop probe is the non-resonating microwave structure and the results can be seen in Figure 5B. It is fed by the RF coaxial cable with a length of 10 cm approx using SMA connectors. The loop probe is not loaded with any reactance elements and the SR is on the same plane.

**Figure 7:**
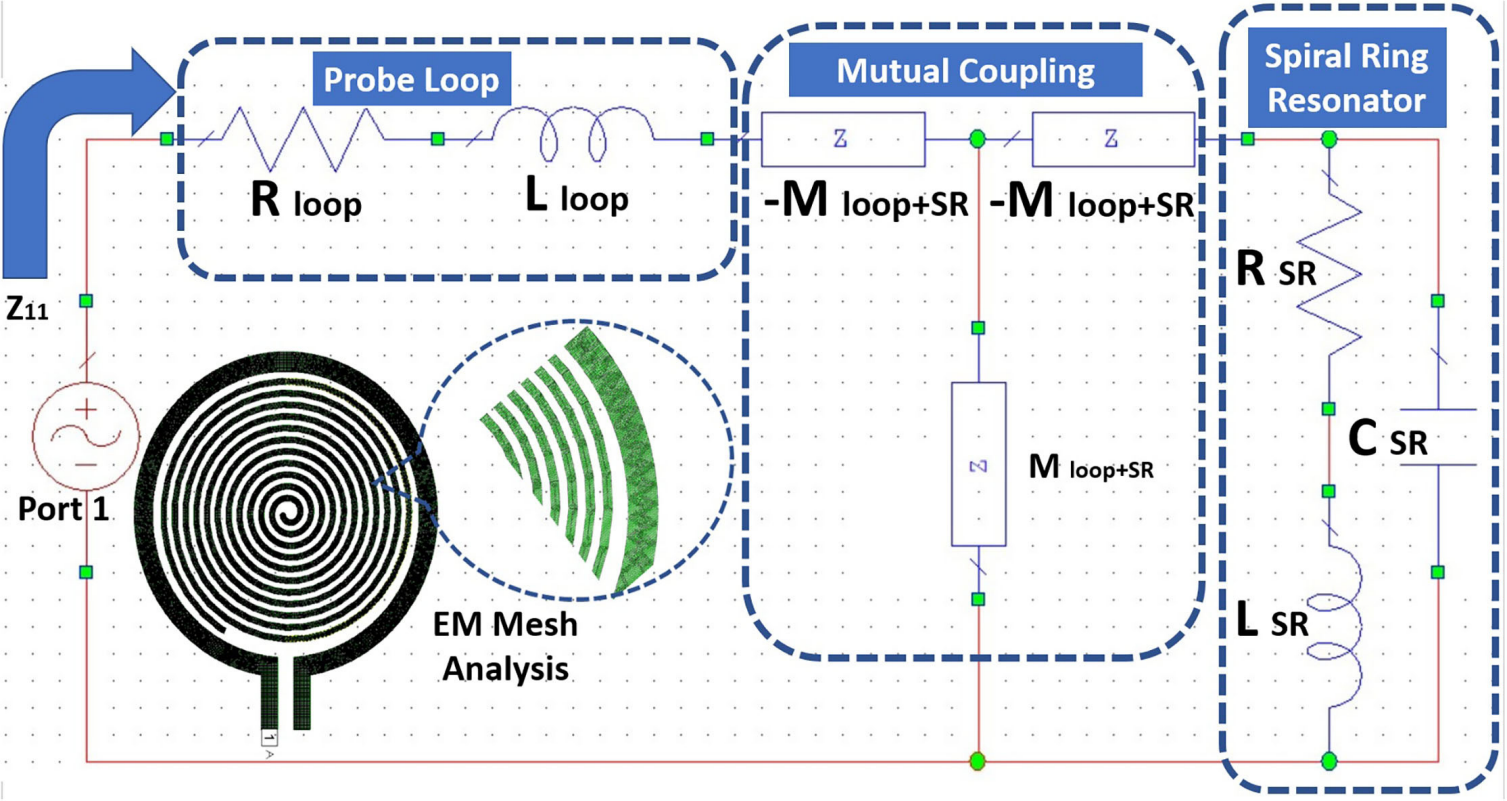
Equivalent RLC circuit and electromagnetic (EM) Mesh analysis for the proposed sensor using SR and probe loop.

The realization of the RLC parameters of the proposed sensors is done using resonant magnetic inclusion as an SR. The estimation of the RLC parameters using a lumped element model is shown in the Figure 7. Also, we have performed a 3D electromagnetic (EM) meshing operation using generic shape-based physical solvers. The SR is represented with RLC elements models as shown in Figure 4. The loop probe is represented with the equivalent circuit, the series resistance R_*loop*_ and the series inductance L_*loop*_. The mutual coupling coefficient M_*loopSR*_ takes into account between the SR circuit and the loop probe circuit.

**Figure 8:**
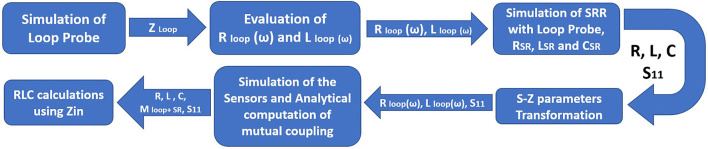
Flowchart of the retrieving RLC parameters for the proposed sensor.

Figure 8 shows the detailed flowchart of the procedure followed to retrieve the RLC parameters of the proposed sensor. The flow starts with the characterization of the standalone loop probe (Figure 6A) using a full-wave EM structure simulator and measured *S*_11_ and input impedance (*Z*_*in*_). The R_*loop*_ (ω) and L_*loop*_ (ω) are made as to the function of frequency (ω), the evaluation is carried out using Z_*in*_ values. The SR in Figure 6B is simulated and S_11_ results are found (48). Then, the sensor is prepared using a loop probe and SR. The PDMS encapsulation is make for the prototype to make the sensor biocompatible.

Now, mutual impedance M_*loopSR*_ needs to be determined using the magnitude of inductive coupling between loop probe and SR. This parameter can be assessed using the magnetostatic method (49) and the geometrical parameters of the sensor are known. Certainly, it is possible to consider the Biot-Savart formulation (6) to estimate the mutual coupling between loop probe and SR. This postulation is validated by the micro dimension of the device concerning wavelength and geometrical structural properties. The geometry of SR is Archimedean spiral, SR linear length is calculated based on the outer diameter, thickness, and number of turns found to be 632 *mm* approx. The resonance frequency of the sensor in the air (Figure 2B) is found to be at 3.37 GHz. SR length is equal to approximately equal 7λ (wavelength at resonance frequency). Also, the resonance frequency of the sensor on the skin is 1.51 GHz and the SR length is equal to 3λ approx. Due to the generic current path in general points, the magnetic field is generated which can be expressed as shown in Equation (6)


(6) $$\begin{array}{l} \underset{B}{\to }\left(\underset{r}{\to }\right)=\frac{\mu_{0}}{4\pi }\int \frac{\left(I\right)\left(\underset{dl}{\to }\right)\times \left(\underset{r^{\prime }}{\to }\right)}{|\underset{r}{\to }{|}^{3}} \end{array}$$

Where μ_0_(H/m) is the magnetic permeability of the vacuum, I(A) is the amplitude of the current, $\underset{dl}{\to }$ is the infinitesimal element of I(A), and $\underset{r^{\prime }}{\to }\left(m\right)$ is the distance dl and generic point in the space. The magnetic flux (ϕ_*ij*_) is defined as the mutual coupling coefficient between loop probe j and SR i through the induced current in SR by current flow in the loop probe. This can be approximated as shown in Equation (7).


(7) $$\begin{array}{l} M_{ij}=\frac{\phi_{ij}}{I_{i}} \end{array}$$

Subsequently, using unit current in the SR (*I*_*i*_), from Equation (6), we can analytically calculate mutual coefficient *M*_*ij*_ (Magnetic flux) through the surface of the loop probe. In this manner, the geometrical properties of the loop probe and SR are specified. We can mathematically set a unit current flowing in either loop probe or SR, and estimate the inductive mutual coupling. Also, it is valid that *M*_*ij*_ = *M*_*ji*_. Therefore, a magnitude of the mutual coefficient expressed in nH can be found. Also, it shows the mutual coupling between loop probe and SR. Consequently, it is possible to estimate *Z*_11_ (*jω*) parameters based on the RLC network as expressed in Equation (8).


(8) $$\begin{array}{l} Z_{11}\left(j\omega \right)=\{[(\left(R_{SR}+j\omega L_{SR}\|\frac{1}{j\omega C_{SR}}\right)\\ \;+\left(-j\omega M_{loopSR}\right))]\|M_{loopSR}\}\\ \;+R_{loop}+j\omega L_{loop}-j\omega M_{loopSR} \end{array}$$

Meanwhile, the RLC parameters related to SR and loop probe are known. Equation (8) is the function that majorly depends on the SR's lumped electric parameters only. It was verified with *S*_11_ characteristics in Figure 2B of the separate tracings of the loop probe and SR.


(9) $$\begin{array}{l} f_{res}≃\frac{1}{2\pi \sqrt{L_{SR}\times C_{SR}}}≃\frac{1}{2\pi }\sqrt{\frac{1}{LC}-{\left(\frac{R_{s}}{L}\right)}^{2}} \end{array}$$

**Figure 9:**
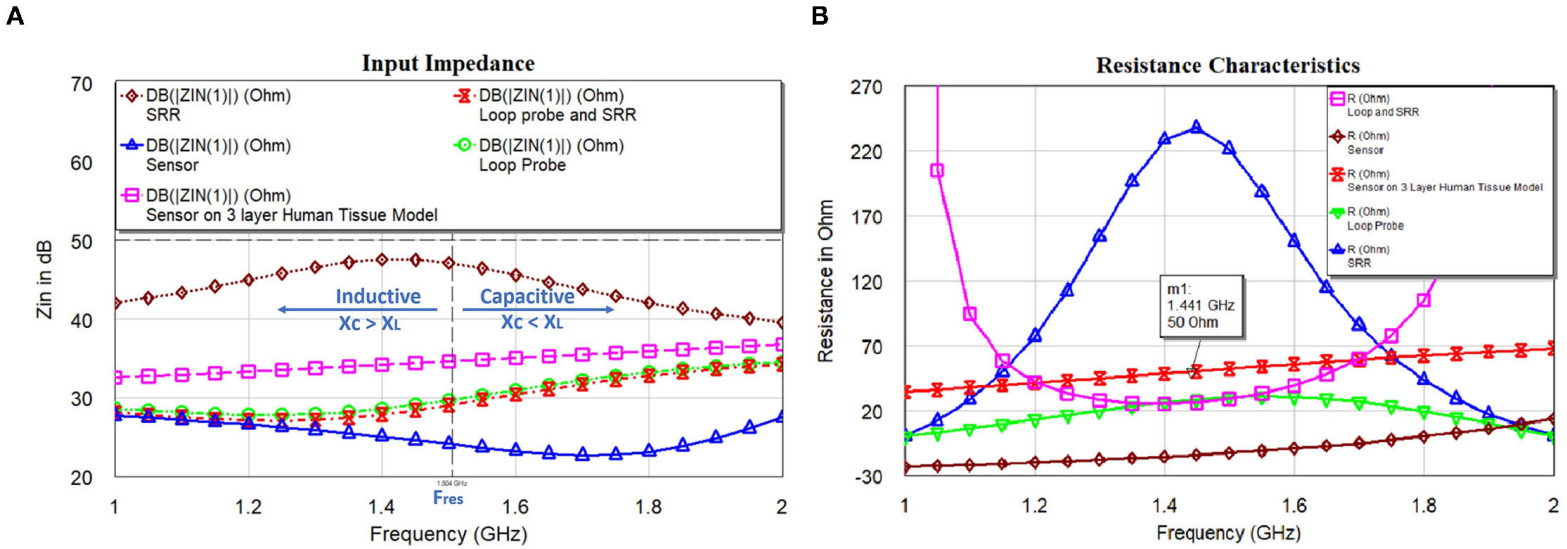
**(A)** Input impedance and **(B)** resistance characteristics of the loop probe (a larger magnetic probe), SR, loop probe with SR, sensor, and sensor on a three-layer human tissue model.

As earlier specified, resonance frequency *f*_*res*_ of the SR is evaluated using the EM structure simulation. This resonance frequency of SR is the function of *C*_*SR*_ and *L*_*SR*_ can be related to the basic resonance Equation (9). The sensor may not be having the characteristics of a pure inductor. So, the approximation of the resonant frequency is made with L: inductance of the sensor; C is the parallel capacitance of the sensor geometry, and *R*_*s*_ is the DC resistive value of the sensor. Alternatively, *R*_*SR*_ influences the Q factor of the SR and extends the appropriate method independently from *C*_*SR*_ and *L*_*SR*_. This way of electrical circuit evaluation will be useful in designing better sensors in the simulation world. Figure 9A shows the input impedance; Figure 9B shows the resistance characteristics of the loop probe, SR, loop probe with SR, sensor, and sensor on a three-layer human tissue model. At the resonance frequency, I(A) will be maximum and circulating between the inductor and capacitor. Similarly, the current distribution in SR will be maximum at the central part due to intern-turn capacitance. The frequency response of the SR can be changed by changing the resistance characteristics. R values affect the flow of the current through the SR if *L*_*SR*_ and *C*_*SR*_ are constant. It is shown in Figure 9B that the sensor is very well matched to 50Ω for the three-layer human tissue model. Near the interested frequency between 1.4 and 1.6 GHz, resistance characteristics is almost near to 50Ω.

The resistance characteristics have a damping effect, based on the proposed equivalent electrical circuit, we can mathematically express as Equation (10). The quantity of damping (50) of RLC resonance is categorized by dampling ratio ζ. Based on our analysis, we considered that the proposed sensor behaves like a parallel resonance circuit. $Z_{n}=\sqrt{\frac{L}{C}}$ is the reactance of every resonating element at the generic points as discussed previously. For the critical damping, ζ = 1, from Equation (10), $R=\frac{Z_{n}}{2}$. Here, resistance in the sensor is achieved by the combination of the copper lines and reactances of the mutual coupling.


(10) $$\begin{array}{l} \zeta ≃\frac{1}{2R}\sqrt{\frac{L_{SR}}{C_{SR}}}≃\frac{Z_{n}}{2R} \end{array}$$

**Figure 10:**
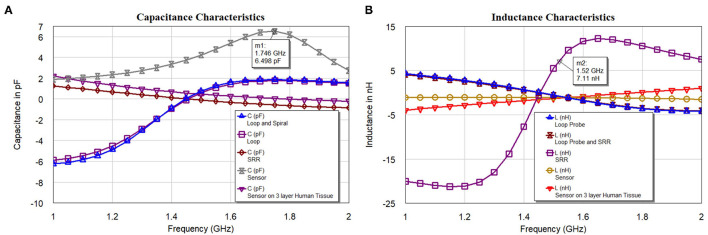
**(A)** Capacitance and **(B)** inductance characteristics of the loop probe (a larger magnetic probe), SR, loop probe with SR, sensor, and sensor on a three-layer human tissue model.

Figure 10A shows the capacitance characteristics and Figure 10B shows the inductance characteristics of the loop probe, SR, loop probe with SR, sensor, and sensor on a three-layer human tissue model. From the proposed equivalent circuit for the sensor (Figure 7), capacitance characteristics (inductive susceptance, *X*_*C*_ = 2π*fC*_*unknown*_) are computed by keeping resistance in parallel. Zin (input impedance) is evaluated and unknown capacitance (*C*_*unknown*_) is measured as expressed in Equation (11) and simulated in the EM structure. The measurement is plotted with real values of capacitance in pF.


(11) $$\begin{array}{l} Z_{in}=R||\frac{1}{j\omega C_{unknown}}=\frac{R\frac{1}{j\omega C_{unknown}}}{R+\frac{1}{j\omega C_{unknown}}} \end{array}$$

Similarly, based on the equivalent circuit, inductance characteristics (inductive susceptance, $X_{L}=\frac{1}{2\pi fL_{unknown}}$) is simulated. The unknown inductance (*L*_*unknown*_) is computed in Figure 10B and mathematically can be calculated using Equation (12).


(12) $$\begin{array}{l} Z_{in}=R+j\omega L_{unknown} \end{array}$$

**Figure 11:**
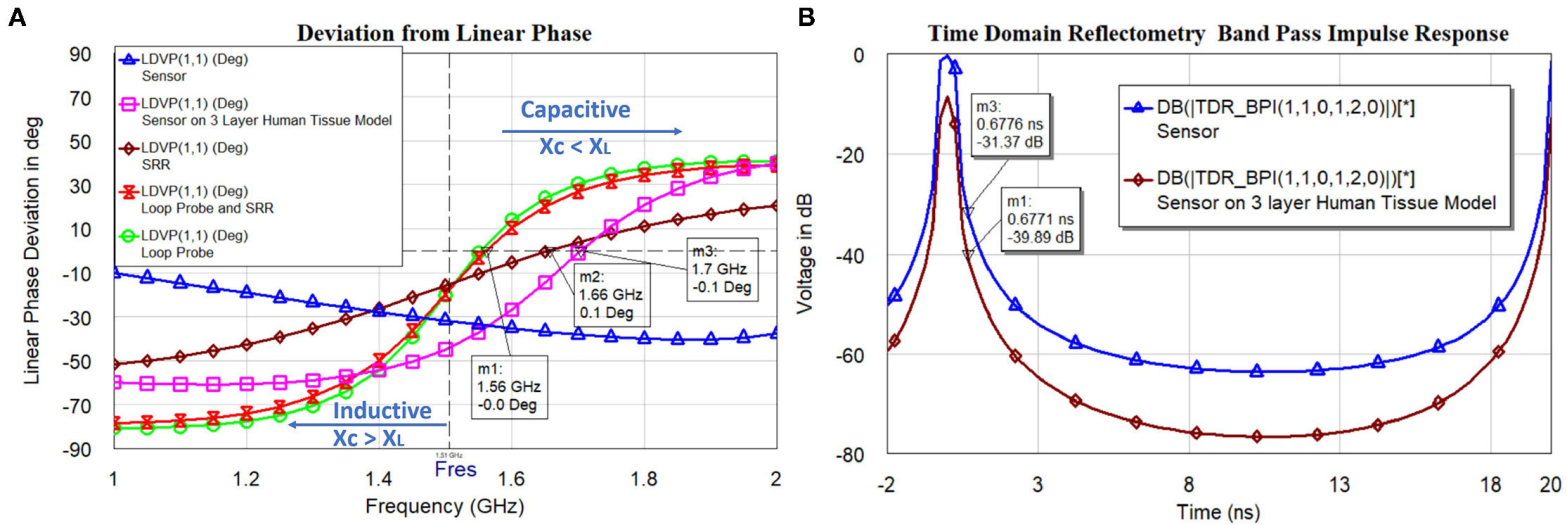
**(A)** The linear phase (DLP) of the loop probe (a larger magnetic probe), SR, loop probe with SR, sensor, and sensor on a three-layer human tissue model, and **(B)** timedomain impulse response of the sensor and sensor on the three layers human tissue model.

The analysis in Figures 10A,B indicates the total susceptance of the circuit reaching almost zero at 1.5 GHz on the three layers human tissue model. This proves that the resonance function is good on the skin. Inductive and capacitive susceptance are having higher values for the sensor in the air. Figure 11A shows the deviation from the linear phase (DLP) of the loop probe, SR, loop probe with SR, sensor, and sensor on a three-layer human tissue model. Based on the proposed equivalent circuit, resonance happens for the input frequency when phase deviation from linear phase is zero, that means zero phase difference between the input voltage and circulating currents producing a resistive circuit.


(13) $$\begin{array}{l} arctan=\frac{X_{L}-X_{C}}{R}=0^{0}deg\left(\forall \mathbb{R}\right) \end{array}$$

Figure 11A shows that DLP is having positive values for the frequencies above the resonant frequency (*F*_*res*_) and negative values for the frequencies below the Fres, which at can mathematically be verified by Equation (13). The measurement is made using the linear simulator (51). DLP varies ±15° maximum for the operation of the sensor on the skin. The λ is related to the electrical length of the SR, so phase deviation occurs in the SR. The Loop probe gets shorter for the higher frequencies. DLP provides information on the linearity of the sensor and distortionless transmission of the microwave in the sensor. The shape (slope) of the curves is proportional to the electrical length of the sensor. In real practice, the sensor is having N turns in the SR and mutual coupling characteristics will delay a few frequencies and result in the non-linear phase shift. The purpose of this analysis is to make sure the sensor on the skin should have the least phase DLP.

Figure 11B shows the simulated time-domain impulse response of the sensor and sensor on the three layers human tissue model. The computed values passed through the bandpass filter (BPF) and Lanczos frequency domain windowing function. The sensor is excited with voltage impulse and time-domain measurement is carried out. Inverse Discrete Fourier Transform (IDFT) mathematical technique is used to transform frequency to the time domain. The simulation is carried between frequency *f*_*start*_ = 1*GHz* and *f*_*stop*_ = 5*GHz* and with *f*_*step*_ = 0.05*GHz*. The alias-free time range $t_{r}=\frac{1}{f_{step}}=20ns$, the x-axis of the plot is up to 20 ns. The alias-free physical distance $d_{r}=\frac{c.t_{r}}{\sqrt{\varepsilon_{r}}}=3.38e^{-4}\left(m\right)$ and physical length of sensor $l=\frac{c}{\sqrt{\varepsilon_{r}}}.\frac{1}{t_{r}}$ for the dielectric constant of the sensor substrate ε_*r*_ = 3 and c is speed of the light. The time resolution or time step (tstep) for the BPF used is calculated by $t_{step}=\frac{1}{f_{stop}-f_{start}}=0.25ns$. This measurement is useful to evaluate faults in the transmission path of the microwaves in the sensor geometry. Also, the voltage difference between the sensor in the air and the sensor on the three-layer human skin model at resonance frequency 1.5 GHz is need to be observed (t = 0.6 ns). The voltage difference found to be 8.5 dB.

In this manner, we can acquire the RLC parameters for the combination of loop probe and SR to the proposed sensor. With the help of EM simulation, we have provided an explicit description of the proposed sensor.

## 3. Results and Discussion

### 3.1. Sensor (*S*_11_) Resonance vs. Dielectric Constant Variation

**Figure 12:**
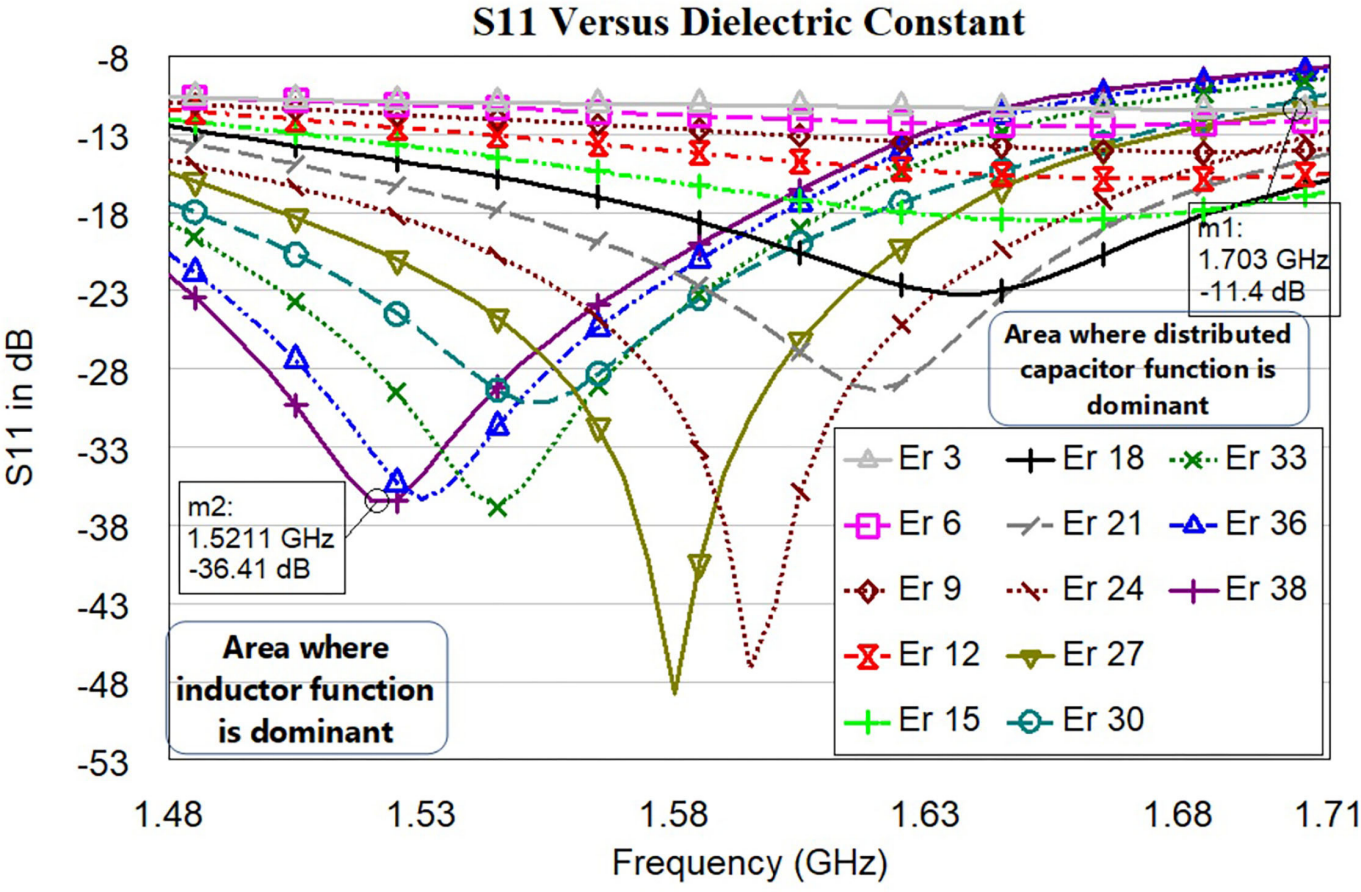
*S*_11_ in dB vs. frequency for the proposed sensor signal response due to dielectric changes of the skin due to burning (ε_*r*_ 3 to 38). Each line color represents the sensor signal response at a different dielectric of the skin.

In this subsection, the operation of the proposed sensor based on the various dielectric constant ε_*r*_ (permittivity) is shown. Figure 12 shows the *S*_11_ in dB vs. frequency for the proposed sensor signal response due to dielectric changes of the skin due to burning (ε_*r*_ 3 to 38). From the analysis made (Figure 1), permittivity varies between ε_*r*_=26 and ε_*r*_=14 for the specific condition (age 30 and normal BMI). The proposed sensor can detect the permittivity between ε_*r*_ 3 and 38 to consider various special cases. The software algorithm will display the degree of burn using change in the resonance frequency shift. The sensor is showing a good response in changing resonance frequency between 1.5 GHz and 1.71 GHz for (ε_*r*_ 3 to 38).

Development of the simulation model to mimic burning tissue yielded useful data regarding the sensor's ability to detect changes of dielectric constant. Dielectric changes in simulation in the three-layer human tissue model triggered shifts in sensor signal response with seven trials being conducted (Figure 12). As the electromagnetic field propagated through the skin and changes of dielectric constant increased, the electromagnetic fields were altered. These alterations prompted shifts in sensor signal response, including a difference in the resonant frequency. Variations in sensor signal response were primarily the result of changes in the effective electric permittivity of the layered system. From the graph, we can understand the changes in the dielectric of skin with respect to resonance frequency. Significant resonance frequency shifts to the left side can be observed by increasing the dielectric value of skin from 3 to 38.

Table 1 shows the relationship between effective permittivity and resonance frequencies. Also, corresponding *S*_11_ values are reported. The numerical data of the resonance frequency and permittivity is taken from Figure 12. The proposed sensor has a good linearity change in permittivity response concerning shift in the resonance frequency. The idea of preparing the sensor is to detect the permittivity by shifting the resonance frequency. We have not considered the values of *S*_11_ as the linear function but it needs to be ≤ −10*dB*. The sensor is working with larger bandwidth between 1.5 GHz and 1.71 GHz, which will make the sensor system efficient. S11 of all resonance frequencies ≤ −11*dB* indicates that the sensor is very well matched with the human tissue model.

**Table 1:** Effective permittivity (ε_*r*_) and resonance frequency relationship.

**Effective permittivity (**ε**_*r*_)**	**Fres (GHz)**	**S11 (dB)**
3	1.71	–11.41
6	1.69	–12.45
9	1.685	–14.14
12	1.675	–15.85
15	1.655	–18.5
18	1.64	–23.21
21	1.62	–29.38
24	1.595	–47.13
27	1.58	–48.8
30	1.55	–30.11
33	1.545	–36.83
36	1.53	–36.37
38	1.525	–36.44

### 3.2. Linear Correlation Between Resonance Frequency vs. Dielectric Constant (ε_*r*_) vs. Burn Degree

**Figure 13:**
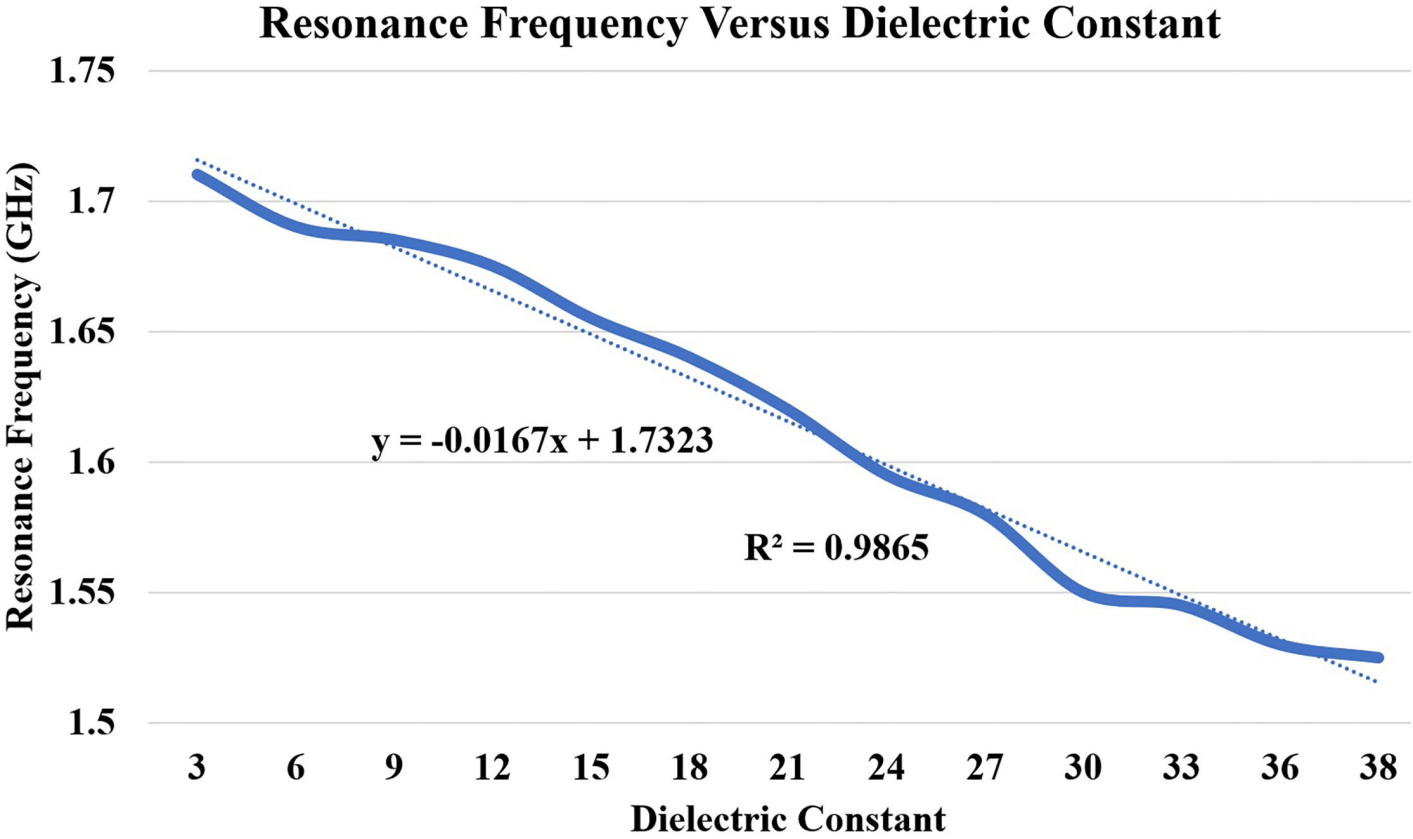
Correlation between dielectric constant (ε_*r*_ 3 to 38) and principal resonant frequency shift (based on *S*_11_). The dotted lines present along with correlated data are the trend lines.

In this subsection, we discuss the correlation between resonance frequency vs. permittivity and burn degree vs. permittivity. Figure 13 shows the relationship between the resonance frequency and permittivity, which is plotted based on the data from Table 1. The dotted line in the figure is linear regression and correlation approximation is made to the sensor data. We found the linear mathematical function expressed in Equation (14). The correlation coefficient (*R*^2^) is found to be 0.98, which indicates the sensor is having a strong correlation to a linear function.


(14) $$\begin{array}{l} F_{res}=-0.0167\varepsilon_{r}+1.7323\wedge R^{2}=0.98 \end{array}$$

From Figure 1, we understood the relationship between permittivity and Burn depth (d), which is almost a linear function. For example, the case discussed in Figure 1 has four different burn degree that seems to be almost linear functions. The linear equations of all different burns are as follows: superficial burn: ε_*r*_ = −0.92*d* + 26; superficial partial-thickness burn: ε_*r*_ = −0.14*d* + 25; deep dermal partial thickness burn: ε_*r*_ = −*d* + 27; and full-thickness: ε_*r*_ = −0.85*d* + 25. The correlation factors algorithm is prepared by using python codes. It is done by considering all possible cases of varying BMI, age, gender, and body parts. As there are more number of clinical trials, the algorithm will be further improved for accurate analysis.

### 3.3. Penetration Depth Analysis Using E-Field Distribution Toward the Tissue

**Figure 14:**
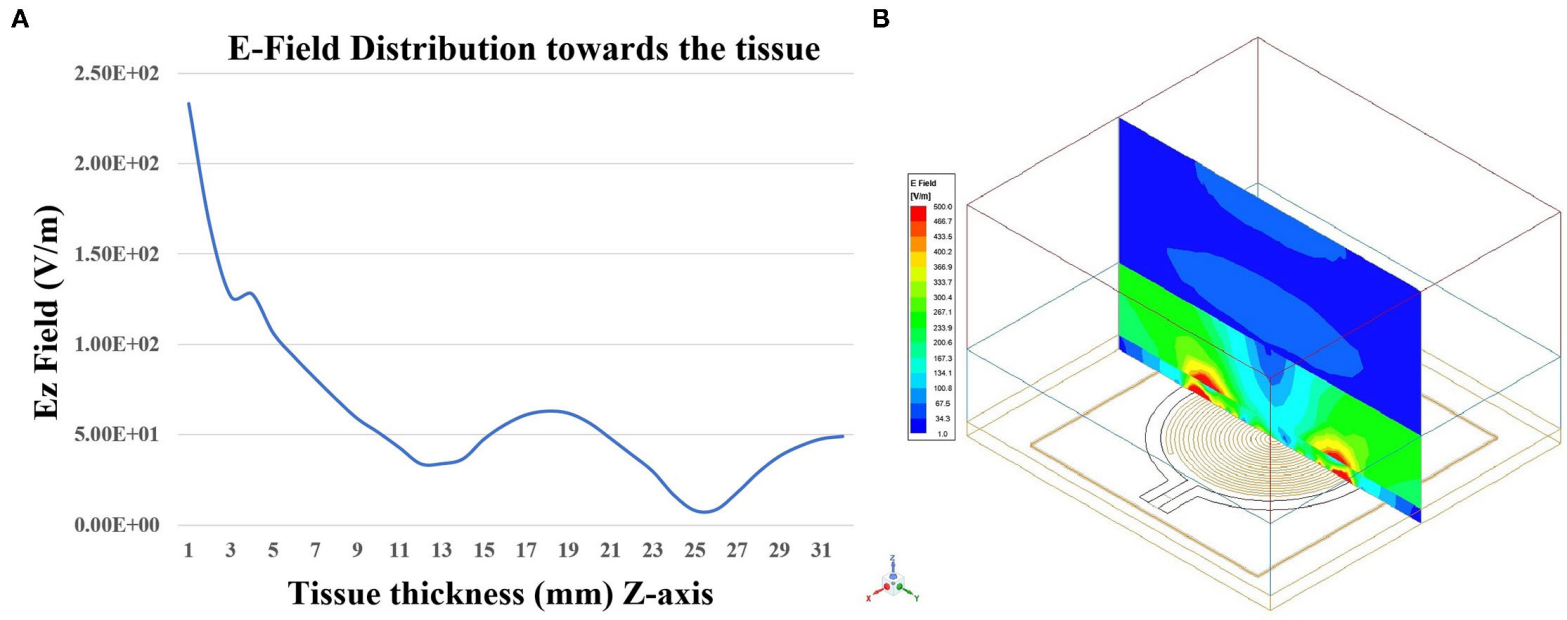
**(A)** Penetration of the *E*_*Z*_-field toward tissue thickness (Z-axis) and **(B)** Isometric 3D view of *E*_*Z*_-field in different layers (skin, fat, and muscle) on the Non-model plane using HFSS software.

The proposed sensor was simulated on the three-layer human tissue model using HFSS software. To investigate the penetration depth, spontaneous analysis of the E-field of the sensor on the three-layered human tissue model was made (Figure 14A). Figure 14B shows the isometric 3D view of Ez-field in different layers (skin, fat, and muscle) on the non-model plane. Figure 14B shows the Ez Field (V/m) values toward the tissue. The sensor placed in the XY plane and Z-axis (perpendicular to the sensor plane) indicates the dimension toward the tissue, which is simulated from 0 to 32 mm. The skin tissue come between 0 and 2 mm, Ez-Field decreases from 2.33 × 10^2^ − 1.26 × 10^2^(*V*/*m*) and fat tissue comes between 2 and 12 mm, Ez-Field values varies between 1.26 × 10^2^ −3.4 × 10^1^(*V*/*m*). The muscle layer lies between the 12 to 32, 3.4 × 10^1^ − 1.8 × 10^1^(*V*/*m*). According to these findings, it can be inferred that microwave signal is penetrating the tissue sufficiently.

### 3.4. Senseburn Sensor System Automation

**Figure 15:**
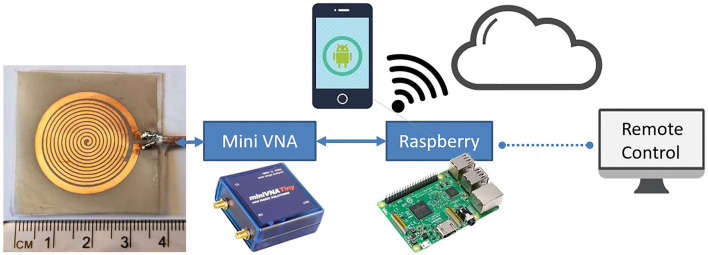
Senseburn system integration using Sensor, MiniVNA, Power Unit, and Raspberry Pi interface.

Figure 15 shows the complete senseburn system integrated using the proposed sensor, MiniVNA, power unit, and Raspberry Pi interface. Regarding the interface between MiniVNA and Raspberry Pi, we have developed the python shell program, which can extract data from MiniVNA, which can be uploaded to the cloud (server) automatically. The system setup consists of a Network analyzer that is connected to the Raspberry Pi on one side and connected from the “device under test” port to a sensor on the other side. This prototype will be used for the upcoming clinical trials.

Two different techniques are used to automate the senseburn sensor system. **(i)** Raspberry Pi is connected wireless with an SH/DNS server. **(ii)** Automated python shell algorithm will be available in the Raspberry Pi for reading, storing, and processing the sensor data.

### 3.5. Latent Medical Applications

The proposed sensor could be utilized to analyze the burn degree for Point-of-Care Applications. The Senseburn sensor is a flexible biocompatible microwave sensor. The proposed technique is a non-invasive diagnosis tool for analyzing the depth and area of a burn injury, with a significant focus on the wound depth evaluation, as none of the current medical devices provides that in real-time in inpatient settings. Since it is a non-invasive diagnostic instrument, it is useful compared to existing techniques like punch biopsy, complete blood count (CBC) measurements, arterial blood gas (ABG), and Human chorionic gonadotropin testing, Serum biochemistry etc. The sensor will be placed on the skin without damaging tissues. The sensor's maximum output power is –6 dBm, and it is minimal power compared to a CT scan (120 kW), which will not be harmful to the tissues. Also, microwave radiation is a form of non-ionizing radiation that is a significant feature compared to X-rays, a form of ionizing radiation. The sensor is designed to quickly detect burn depth up to 3 cm, sufficient to analyze all degrees of burn. This sensor is used to observe the penetration based on different parameters such as resonance frequency (*F*_*res*_), effective permittivity ε_*r*_, and tissue thickness ts. Furthermore, the signal that penetrated through different tissue layers was investigated in terms of its E-field to analyze the signal penetration depth. The sensor is connected to MiniVNA and scanned initially on healthy skin and later scanned on the burnt areas. Based on the change in resonance frequency fr and physical parameters like age, gender, landmark and BMI, burnt depth will be evaluated. The process is automated using Raspberry Pi and graphical user interface activated in the cloud.

## 4. Conclusion

The proposed EM resonator-based non-invasive flexible biocompatible sensor is developed based on the numerical simulation and it could detect the changes of permittivity variations of the burnt human skin. The sensor is having a sensitive variation of the permittivity (ε_*r*_ 3 to 38) with the indication of resonance frequency shift between 1.5 and 1.71 GHz. The physical dimension of the sensor is 34 x 39 x 1.4 mm. To make the sensor biocompatible, we have encapsulated the sensor with quality PDMS. The sensor is characterized using EM simulations and an electrical equivalent model using RLC parameters. We have provided mathematical and analytical realizations of the sensor. We have analyzed the capacitance, inductance, resistance, and impedance characteristics of the sensor. Also, the analysis includes phase deviation from linear phase and time domain reflectometry simulations. Consequently, we obtained an accurate analysis of the EM properties of the sensor. The software algorithm is prepared to correlate the permittivity variation with burn depth to the sensor resonance frequency characteristics. We have designed the compact, portable, and user-friendly senseburn system suitable for clinical application.

Table 2 shows the comparison of the proposed sensor results with previously published work. In this comparison, we have considered only a SR with a loop probe-based sensor. Also, we considered the sensor which works similar to the proposed sensor technique. Based on this comparison, this is the new attempt made to design at 1.5 GHz and provided a very good sensing bandwidth of 1.5–1.71 GHz. Additionally, the size of the proposed sensor is smaller (one-third) than the other mentioned sensor. Finally, the proposed sensor is bio-compatible, flexible, and good sensing bandwidth for quality performance. The developed flexible sensors in this work will provide better health care for the patients and also will provide user comfort at the time of measurement. The development of hive sensors required synchronized data collections of various locations of the human body.

**Table 2:** Comparison of the proposed sensor performance with previously published work.

**References**	**Fres**	**Bandwidth**	**Size (L x W x T)**	**Remarks**
Cluff et al. (17)	3.9 MHz	1.1–25 MHz	10 cm x 10 cm x 0.2 mm	EM resonator detect the relationship between resonant frequency and fluid volume (0–100 ml)
Woodard (52)	17.1 MHz	15–35 MHz	10 cm x 10 cm x 0.13 mm	Self-resonating planar sensor detects the fluid level
Alruwaili et al. (53)	3 MHz	1.4–1.48 MHz	10 cm x 10 cm x 0.48 mm	Volumetric sensitivity Measurement (20–100 ml) with temperature parameter
Kostogorova-Beller et al. (54)	8.2 MHz	8–8.6 MHz	7.6 cm x 7.6 cm x 0.78 mm	SansEC sensor detect deflection speed
This Work	1.5 GHz	1.5–1.71 GHz	34 mm x 39 mm x 1.4 mm	Sensor detects the change in dielectric property variation of the burnt skin

In the future, the performance of the sensor will be evaluated with various phantoms and on *ex-vivo* human burnt skin samples. Later, the sensor will be used in clinical trials on burn patients before the surgery. Eventually, an extensive database will be prepared with various burn conditions of the skin. This work will be optimized based on the clinical trials and clinician feedback.

## Data Availability Statement

Publicly available datasets were analyzed in this study. This data can be found at: http://niremf.ifac.cnr.it/tissprop/.

## Author Contributions

PR performed RLC estimation of the proposed, prepared conceptual correlating software algorithm of the senseburn device, and wrote most of the article. BM designed the sensor, tested its performance and was involved in the critical analysis of the sensor working. EA was involved in the simulation of the permittivity vs. burn degree algorithm. AC prepared the compact hardware for the senseburn system and was involved in the hardware part of the sensor. BA has done the review, proofreading, and provided techniques related to flexible circuit printing. MP wrote the introduction, discussion, and was actively involved in all the steps of the senseburn system. RA was involved in the supervision of the project, conception, design of the research, and approved the final version of the manuscript. All authors contributed to the article and approved the submitted version.

## Funding

This work was supported by the following Projects: Senseburn by Eureka Eurostar (2018/410), Swedish Vinnova Project BDAS (2015-04159), Swedish Vetenskapradet (VR) Project Osteodiagosis (2017–04644), and Swedish SSF Project LifeSec (RIT170020).

## Conflict of Interest

The authors declare that the research was conducted in the absence of any commercial or financial relationships that could be construed as a potential conflict of interest.

## Publisher's Note

All claims expressed in this article are solely those of the authors and do not necessarily represent those of their affiliated organizations, or those of the publisher, the editors and the reviewers. Any product that may be evaluated in this article, or claim that may be made by its manufacturer, is not guaranteed or endorsed by the publisher.
